# Successful use of a hepatitis C viremic donor in pediatric bilateral lobar lung transplantation

**DOI:** 10.1016/j.xjtc.2022.06.008

**Published:** 2022-06-18

**Authors:** Mitsuaki Kawashima, Elias Seidl, Hartmut Grasemann, Seyed Alireza Rabi, Terunaga Inage, Kazuhiro Yasufuku, Shaf Keshavjee, Jordan J. Feld, Marcelo Cypel

**Affiliations:** a Division of Thoracic Surgery, Department of Surgery, Toronto General Hospital, University Health Network, University of Toronto, Toronto Lung Transplant Program, Toronto, Ontario, Canada; b Division of Respiratory Medicine, Department of Pediatrics, The Hospital for Sick Children, Toronto, Ontario, Canada; c Toronto Centre for Liver Disease, Toronto General Hospital, University Health Network, Toronto, Ontario, Canada

**Figure:**
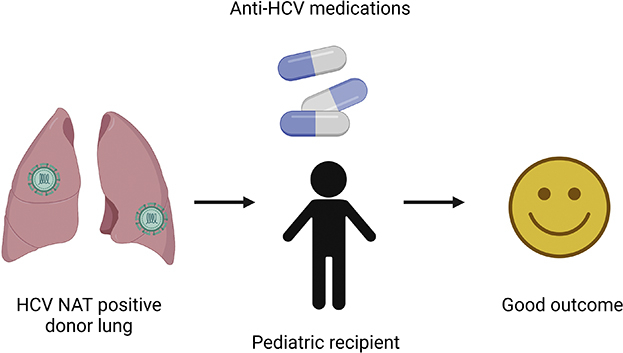
Strategy to use HCV NAT–positive donor lung in pediatric patients.

**Central Message**

We report a first transplant using HCV NAT–positive donor lung to a pediatric patient with short-course anti-HCV agents. This strategy can address donor lung shortage for pediatric recipients.

Reduced availability for properly sized donors can result in longer wait times for children compared with adults awaiting lung transplantation (LT). Increasing the donor pool for listed pediatric patients is therefore a priority. Lungs from donors infected with hepatitis C virus (HCV) have been used successfully for transplantation in adults using antiviral agents immediately in the recipients to prevent chronic HCV infection.^1^^,^^2^ Here, we report the first case of a pediatric bilateral lobar LT from an HCV nucleic acid amplification testing (NAT)-positive donor. The subject provided informed written consent for the publication.

## Case Report

**Figure 1:**
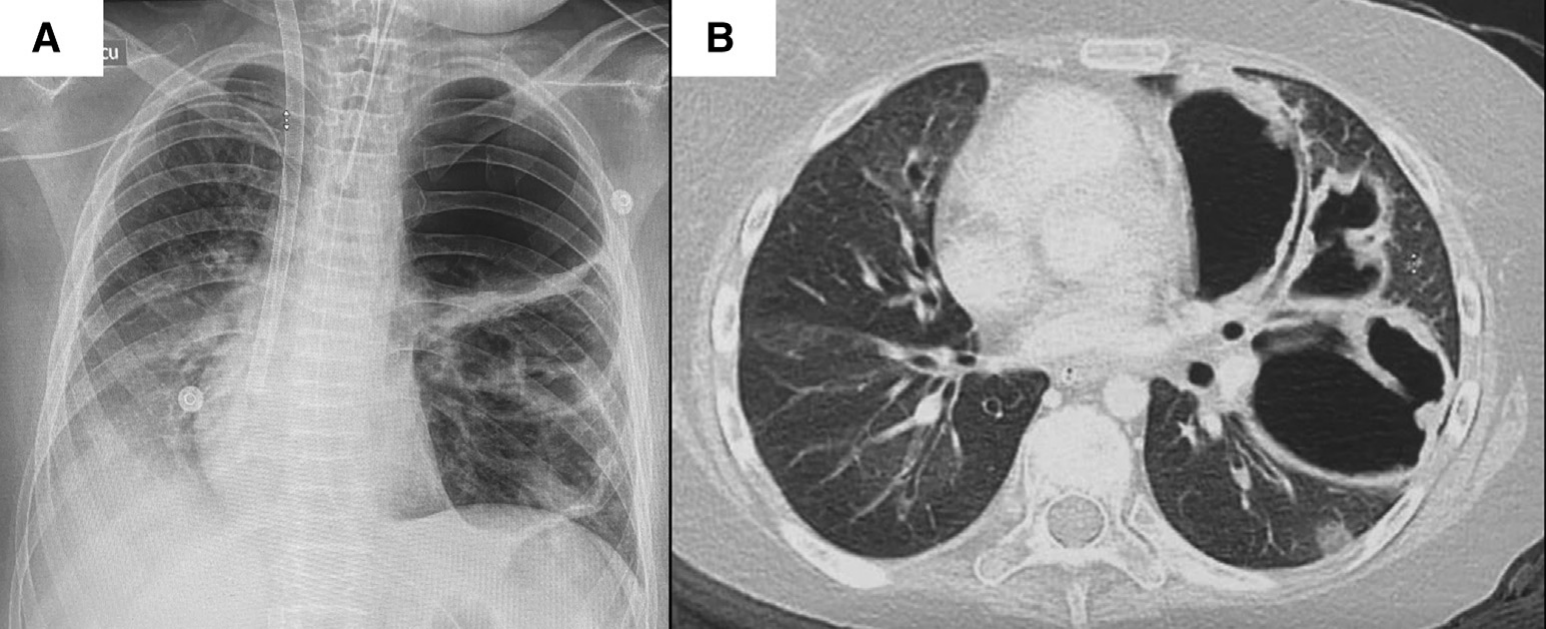
Preoperative radiograph and computed tomography scan. A, Preoperative radiograph shows large cavitary lesion at left upper lung zone, mediastinal shift to the right and peripheral VV ECMO cannulas. B, Preoperative computed tomography shows bronchiectasis throughout the lung, mosaic attenuation of the lung parenchyma, and large cavitary lesion at left upper lobe.

A 13-year-old girl with a medical history of chronic graft versus host disease and bronchiolitis obliterans following bone marrow transplantation at 9 years of age for acute myeloid leukemia was listed for LT, when she became hypercapnic (partial pressure of carbon dioxide 75 mm Hg) and was started on supplementary oxygen during the day (3.5 L/min) and noninvasive ventilation during sleep. Her forced expiratory volume in 1 second was 17% of predicted (0.43 L), forced vital capacity was 1.48 L (53% predicted), and total lung capacity was 3.47 L. Radiographically, there were bronchiectatic changes throughout the lungs, mosaic attenuation, and multiple thick-walled cavitating lesions within the left upper lobe (Figure 1). Ventilation/perfusion scan showed differential perfusion 41% and 59% of the right and left lung, respectively. The patient was 150 cm tall, her weight was 51 kg, body mass index 24 kg/m^2^, and her blood type was A positive. Serologically, she was negative for HCV antibodies at listing for LT.

A month after listing, the patient deteriorated requiring endotracheal intubation and cannulation for venovenous extracorporeal membrane oxygenation (VV ECMO) as a bridge to LT. A potential donor became available, who was an adult in his/her thirties with unrecoverable hypoxic brain damage due to overdose. The donor was 173 cm, 58 kg, predicted total lung capacity 6.07 L, blood type A positive, and tested positive for HCV NAT. The patient's family consented after being informed about the HCV NAT positivity and our anti-HCV protocol, which uses ezetimibe and glecaprevir–pibrentasvir. In Canada, ezetimibe is approved for children 10 years old and older, and glecaprevir–pibrentasvir is approved for children 3 years old and older. The first dose of ezetimibe (10 mg) to block HCV entry in hepatocytes and glecaprevir–pibrentasvir 300 mg/120 mg to block HCV replication was given to the recipient right after the procurement of the donor lung, which was 6 hours preoperatively to the recipient procedure.

**Figure 2:**
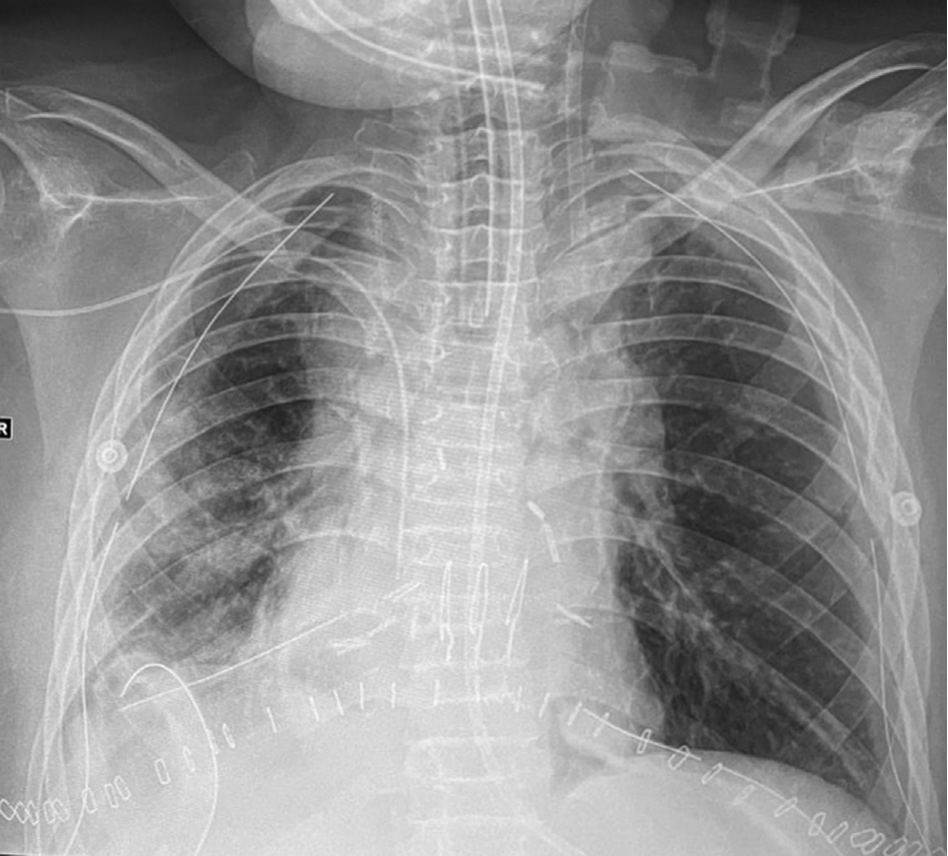
Postoperative radiograph. Postoperative radiograph taken on postoperative day 2 shows good-size matching and aeration of both lung grafts.

The recipient was brought to the operating room with pre-existing VV ECMO set up. Given the hemodynamic stability and absence of pulmonary hypertension, we decided to proceed with LT on VV ECMO. Bilateral lobar LT was performed using both lower lobes as described elsewhere.^3^ At the end of the procedure, she was successfully weaned from VV ECMO and transferred back to the pediatric intensive care unit. The initial postoperative partial pressure of oxygen was 152 mm Hg with fraction of inspired oxygen (Fio_2_) 1.0 and positive end-expiratory pressure 8 cm H_2_O. The partial pressure of oxygen/Fio_2_ ratio obtained on postoperative day (POD) 1 was 302 mm Hg with Fio_2_ 0.4 and positive end-expiratory pressure 8 cmH_2_O. Radiograph of the chest showed good size match and aeration (Figure 2).

Glecaprevir–pibrentasvir and ezetimibe were given once daily for an additional 7 days postoperatively. The patient was transferred from the pediatric intensive care unit to the transplant ward on POD 14. She got discharged to a rehabilitation facility on POD50 and has been doing well.

Her HCV viral titer was monitored on day 1, day 7, day 14, 1 month, 2 months, and 5 months posttransplant, which has revealed no detectable viremia up to 5 months postoperatively at the timing of writing.

## Discussion

Lungs from HCV-positive donors have been successfully transplanted for adult recipients treated with anti-HCV medications pre- and postoperatively.^1^^,^^2^ Our group has reported excellent outcomes with ultrashort course ezetimibe (HCV entry blocker) and glecaprevir–pibrentasvir (HCV replication inhibitor) in adult LT recipients.^1^ However, there have been no reports to date regarding the use of HCV NAT–positive donors for pediatric solid-organ transplantation except for a report regarding usage of HCV seropositive heart to children.^4^ Our case highlights the safety and feasibility of our protocol for pediatric LT recipients.

Pediatric patients often encounter long waiting times because of scarcity of pediatric or size-matched donors. To overcome donor shortage for patients with small size, lobar LT from bigger sized donors is an option that is also applicable for adult recipients with smaller size. We previously reported outcomes of lobar LT with satisfactory outcomes.^5^ To conclude, usage of HCV NAT–positive donor combined with bilateral lobar LT from a donation after cardiac death donor was a feasible strategy for a critically ill pediatric patient.
